# M. Dumeril on the Preservation of the Nitrate of Silver

**Published:** 1842-11-05

**Authors:** 


*Preservation of Nitrate of Silver.* By M. Dumeril.—For this purpose M. Dumeril has invented a plan. He melts in a vessel over a fire the best sealing-wax, containing a large quantity of gum-lac, and in this he dips the piece of caustic, over which he thus obtains a complete and firmly adherent varnish impermeable by the air or light, and completely preventing the staining of the fingers. In using the caustic, all that is necessary is to scrape off with a penknife as much of the wax as is required to expose a surface of given size. Caustic thus coated is of course very convenient for introducing into cavities of which only a small portion is to be touched.—*Ibid, from Bul. Gén. de Thérapeutique*, Mai, 1842.

